# Gamma-Aminobutyric Acid Promotes Beige Adipocyte Reconstruction by Modulating the Gut Microbiota in Obese Mice

**DOI:** 10.3390/nu15020456

**Published:** 2023-01-15

**Authors:** Xiaoyi Ma, Huanhuan Yan, Shubin Hong, Shuang Yu, Yingying Gong, Dide Wu, Yanbing Li, Haipeng Xiao

**Affiliations:** 1 Department of Endocrinology, The First Affiliated Hospital, Sun Yat-Sen University, Guangzhou 510080, China; 2 Paul C. Lauterbur Research Center for Biomedical Imaging, Shenzhen Institutes of Advanced Technology, Chinese Academy of Sciences, Shenzhen 518055, China; 3 Department of Geriatrics, The First Affiliated Hospital, Sun Yat-Sen University, Guangzhou 510080, China

**Keywords:** obesity, GABA, white adipose, beige adipose, gut microbiota

## Abstract

Given the increasing prevalence of obesity, the white-to-beige adipocyte conversion has attracted interest as a target for obesity treatment. Gamma-aminobutyric acid (GABA) treatment can reduce obesity, but the underlying mechanism remains unclear. Here, we aimed to investigate the mechanism by which GABA triggers weight loss by improving the beiging of inguinal white adipose tissue (iWAT) and the role of gut microbiota in this process. The results showed that GABA reduced body weight and adipose inflammation and promoted the expression of thermogenic genes in the iWAT. The 16S rRNA sequence analysis of gut microbiota showed that GABA treatment increased the relative abundance of Bacteroidetes, Akkermansia, and Romboutsia and reduced that of Firmicutes and Erysipelatoclostridium in obese mice. Additionally, serum metabolomic analysis revealed that GABA treatment increased 3-hydroxybutyrate and reduced oxidized lipid levels in obese mice. Spearman’s correlation analysis showed that Akkermansia and Romboutsia were negatively associated with the levels of oxidized lipids. Fecal microbiota transplantation analysis confirmed that the gut microbiota was involved in the white-to-beige adipocyte reconstruction by GABA. Overall, our findings suggest that GABA treatment may promote iWAT beiging through the gut microbiota in obese mice. GABA may be utilized to protect obese people against metabolic abnormalities brought on by obesity and gut dysbiosis.

## 1. Introduction

The incidence of obesity has risen at an unprecedented rate over the past 30 years, seriously endangering human health and socio-economic development [1,2]. The most common way to lose weight is through lifestyle interventions, such as exercise and diet, but these are difficult for patients to maintain. Most weight loss drugs on the market, meanwhile, have undesirable side effects [3]. Previous studies have shown that the beiging of inguinal white adipose tissue (iWAT) facilitates weight loss and has an anti-obesity effect [4,5]. Therefore, a strategy for increasing beige fat activity could be an effective therapeutic for preventing obesity.

It was previously believed that adipose tissue could be divided into two categories—white and brown adipose. Brown adipose tissue (BAT), which is an important thermogenic tissue in the body, contains uncoupling protein 1 (UCP1) and higher numbers of mitochondria. Meanwhile, WAT plays a major role in lipid and energy storage. In recent years, beige adipose, a third type of adipose tissue, has been identified, which can also facilitate thermogenesis and energy expenditure, thereby promoting weight loss [6,7,8]. Previous studies have shown that chronic cold stimulation, long-term usage of peroxisome proliferator activated receptor agonists, and other relevant interventions can all promote the beiging of WAT [4]; however, these approaches are not feasible.

The gut microbiota play an important role in the human body and is regarded as a “hidden organ” [9]. Gut microbiota can play an important role in regulating beige fat tissue and non-shivering thermogenesis [10,11,12,13,14]. Furthermore, previous studies have shown that the transfer of “cold” bacteria to germ-free mice can increase the rate of beige adipose reconstruction and improve energy consumption [15,16]. Gut microbiota may be an important area of focus for losing weight and preventing obesity. Gamma-aminobutyric acid (GABA) has been investigated the important role in glucose metabolism [17,18]. Researchers have found that GABA treatment can improve the inflammatory response, restore balance to the gut microbiota of obese mice, and thus play a role in regulating metabolism [19,20]. Our previous study reported that GABA promotes weight loss and improves glucose metabolism in high-fat diet (HFD)-fed mice [17]. However, the underlying mechanism remains unclear.

Based on the above-mentioned considerations, this study aimed to examine the effects of GABA in HFD-fed obese mice and to investigate the composition of gut microbiota via 16S rRNA sequencing. Furthermore, we performed an integrative analysis of the relationships between gut microbiota and serum metabolites to understand the potential anti-obesity mechanism of GABA. Our findings will inform therapeutic strategies for obesity and obesity-related related disorders.

## 2. Materials and Methods

### 2.1. Animal Experiments and Study Design

Male C57BL/6J mice (six weeks old) were purchased from HFK Bio-Technology Co. Ltd. (Beijing, China). The mice were housed in a temperature- and humidity-controlled environment with a 12-h light/dark cycle at a room temperature of 22 ± 2 °C and a humidity of 55 ± 5%. They were given free access to water and food.

Mice were fed either a standard diet (SD, D12450B, 10% fat) or an HFD (D12492; Research Diets, 60% fat, 20% protein, and 20% carbohydrate). After 12 weeks, the mice were randomly divided into four groups and fed one of the following diets: (1) SD without GABA treatment (SD), (2) SD with GABA treatment (SD+GABA), (3) HFD without GABA treatment (HFD), or (4) HFD with GABA treatment (HFD+GABA). The two groups of GABA-treated mice received 6 mg·mL^−1^ GABA (A5835, Sigma-Aldrich, St. Louis, MO, USA) in their drinking water for a period of 4 weeks, according to the methods described in our previous study [17].

### 2.2. Glucose and Insulin Tolerance Test

Fasting blood glucose (FBG) levels were measured in the blood samples collected from tail veins after fasting the mice for 16 h. Glucose tolerance tests (GTT) and insulin tolerance tests (ITT) were conducted at the beginning and end of animal treatment, according to previously published methods [21]. Briefly, the GTT was performed with a dose of 2 mg·kg^−1^ of glucose per body weight; blood glucose was measured before and after 30, 60, and 120 min of intraperitoneal injection of glucose. Similarly, ITT was performed with an insulin dose of 0.75 U·kg^−1^; blood glucose was measured at 0, 15, 30, 60, and 90 min after insulin injection.

### 2.3. Cold Stimulation Test

Before performing cold stimulation, the rectal temperature of each mouse at room temperature (25 °C) was recorded as a measure of the basal body temperature (at 0 min); mice were then placed under cold storage conditions (4 °C). The rectal temperature of the mice was measured and recorded after 30, 60, 120, and 180 min. Water and food were not provided during the test. After the experiment, the mice were transferred to room temperature as soon as possible following which they were free to eat and drink water.

### 2.4. Tissue Collection and Immunostaining

Mice were euthanized via cervical dislocation, following which small intestine, colon, iWAT, epididymal WAT (eWAT), and BAT samples were immediately collected. Samples were then quickly placed in a freezer at −80 °C until use. Subsequently, the samples were harvested and fixed in 4% formaldehyde overnight for histological examination.

Paraffin-embedded small intestine, colon, and iWAT sections (5 μm) were stained with hematoxylin and eosin (H&E) or periodic acid Schiff (PAS) [14]. For immunohistochemical (IHC) staining, the primary IHC antibodies against UCP1 (ab10983, 1:1000), CD86 (ab119857, 1:1000), and ZO-1 (abG041, 1:1000) were purchased from Abcam (Cambridge, UK). Images of tissue samples were acquired using an Olympus BX53 microscope (Tokyo, Japan). Image-Pro Plus v.6.0 software was used to analyze adipocyte diameters in the H&E-stained sections.

### 2.5. Gut Microbiota Analysis

The gut microbiota was analyzed following a previously published method [22]. Briefly, at the end of the above experiment, fecal samples were collected from each mouse and stored at −80 °C. Genomic DNA was extracted from fecal samples using the QIAamp DNA stool kit (Qiagen, Hilden, Germany), following which the purity and concentration of DNA were determined via agarose gel electrophoresis. The V3-V4 region of the bacterial 16S ribosomal ribonucleic acid (rRNA) gene was amplified using polymerase chain reaction (PCR); the PCR product was identified with agarose gel electrophoresis (2%), and the target band was recovered. The TruSeq^®^ DNA PCR-Free sample preparation kit (Illumina, San Diego, CA, USA) was used to construct the library. After the library was qualified, online sequencing was performed usingIllumina NovaSeq6000 (Otogenetics, Norcross, GE, USA). The sequences clustered with 97% similar taxonomy were grouped as an operational taxonomic unit (OTU). The free online Metware Cloud Platform (Metware Biotechnology Co., Ltd., Wuhan, China) was used to conduct linear discriminant analysis (LDA) effect size (LEfSe), Chao 1, Shannon index and non-metric multidimensional scaling (NMDS), and Tax4Fun.

### 2.6. Non-Targeted Metabolomics

Serum collected at the end of the study period was used for non-targeted metabolomic data analysis. Serum was isolated from the whole blood collected from anesthetized mice via retro-orbital sampling. In accordance with the manufacturer’s instructions, the serum samples were placed on ice, three times the volume of ice-cold methanol was added, and the samples were centrifuged for 10 min (12,000 rpm, 4 °C). Then, the supernatant was collected and centrifuged for 5 min (12,000 rpm, 4 °C). Finally, the supernatant was collected and used for liquid chromatography with tandem mass spectrometry analysis (UPLC, Shim-pack UFLC SHIMADZU CBM A system, https://www.shimadzu.com/ 13 November 2022, Shimadzu, Columbia, OR, USA; MS, QTRAP^®^ System, https://sciex.com/ 13 November 2022, Sciex, Framingham, MA, USA), according to standard protocols. The Metware Cloud Platform (Metware Biotechnology Co., Ltd., Wuhan, China) was used for metabolomics data analysis.

### 2.7. Magnetic Resonance Imaging (MRI)

After 16 weeks, MRI was used to assess visceral and subcutaneous fat in mice fed with the SD or an HFD. Images were acquired using a 9.4 Tesla, 30 cm-diameter-bore small animal MRI scanner (uMR 9.4T, United Imaging Life Science Instrument Co., Ltd., Wuhan, China) equipped with a gradient insert with a maximum strength at 1000 mT/m and a slew rate up to 10,000 T/m/s. For homogenous total-body scanning, a two-channel volume coil with an 86 mm inner diameter was used for both transmitting and receiving signals. T1 weighted multi-slice fast spin echo scans were performed on the entire mouse body horizontally; the parameters were as follows: TE/TR = 8.72/500 ms, BW = 360 Hz/pixel, field of view: 40 × 96 mm^2^, data matrix size 426 × 1024, 22 × 0.5 mm slices with a 0.1 mm slice gap. Data were analyzed using Carimas software (version 2.9, Turku PET Centre, Turku, Finland).

### 2.8. Fecal Microbiota Transplantation (FMT)

For in vivo antibiotic treatment, mice fed with an HFD at the 12th week were treated with combined antibiotics (Abx), containing 100 U·mL^−1^ penicillin, 50 µg·mL^−1^ vancomycin, 100 µg·mL^−1^ neomycin, 50 µg·mL^−1^ streptomycin, 100 µg·mL^−1^ metronidazole, 10 µg·mL^−1^ chloramphenicol, 1 mg·mL^−1^ bacitracin, 125 µg·mL^−1^ ciprofloxacin, and 170 µg·mL^−1^ gentamycin, for 5 days, according to previously published methods [10]. Then, mice were randomly divided into three groups based on the obese mouse model: non-antibiotics-treated group (HFD/Vehicle), antibiotics/vehicle-treated group (Abx/Vehicle), and antibiotics/GABA-treated group (Abx/GABA). The mice of Abx/GABA group received 6 mg·mL^−1^ of GABA (A5835, Sigma-Aldrich, St. Louis, MO, USA) in their drinking water for 4 weeks.

For the FMT experiment, the antibiotics-treated obese mice were randomly divided into HFD/FMT and GABA/FMT group. After treatment with antibiotics, the fecal microbial supernatant from the HFD group and the HFD+GABA group was gavaged into the HFD/FMT and GABA/FMT mice (150 μL·d^−1^ for 7 consecutive days, with 0.1 g feces/mL), respectively, according to a previously reported method [23]. The body weights and FBG of all mice were recorded before and 4 weeks after FMT.

### 2.9. Quantitative PCR Analysis

Total RNA was extracted from iWAT and BAT samples using a quantitative PCR kit (TRIzol reagent, Taraka, Kyoto, Japan), according to the manufacturer’s instructions. To synthesize the complementary DNA (cDNA), total RNA was reverse-transcribed using a cDNA Synthesis Kit (TaKaRa, Kyoto, Japan). Real-time PCR amplification was performed using gene-specific primers and a SYBR Green real-time PCR kit (Takara, Kyoto, Japan). The gene primers used in this study are listed in Table 1. The expression levels of the target gene were normalized to those of β-actin in the cDNA sample; they were calculated using the 2^−△△CT^ statistical method.

### 2.10. Western Blot Analysis

Western blot analysis of tissues was performed following standard procedures described in a previously published paper [24]. Loading controls were established using β-actin immunoblots. Briefly, for each sample, 40 mg tissue lysate was separated using 10% sodium dodecyl sulfate-polyacrylamide gel electrophoresis, following which the resolved proteins were transferred onto polyvinylidene fluoride (PVDF) membranes. The PVDF membranes were incubated with a blocking buffer (5% skimmed milk) for 1 h and then incubated with the following primary antibodies: anti-UCP1 (1:2,000, Abcam, ab10983) and anti-β-actin (1:5000, CST, #4937). These antibodies were diluted in the blocking buffer overnight at 4 °C. The next day, the membranes were washed and incubated with appropriate secondary antibodies for 1 h at room temperature. Finally, the PVDF membranes were washed thrice, and the protein signal was detected using an enhanced chemiluminescence reagent (Pierce, Rockford, IL, USA). Immunoreactive proteins were then detected using an enhanced chemiluminescence system (GE Healthcare Life Sciences, Buckinghamshire, UK). Western blots were quantified with the Image J software (NIH, Bethesda, MD, USA), using β-actin as an internal control.

### 2.11. Statistical Analysis

All data are reported as mean ± standard deviation. Statistical differences were analyzed using a two-tailed Student’s *t*-test or analysis of variance (ANOVA); the Tukey-Kramer test was used as a multiple comparison post-hoc test. A *p*-value < 0.05 was considered significant. Differences in the LEfse ratios of core fecal microbiota were analyzed using the non-parametric Kruskal-Wallis rank sum test. Spearman’s correlation was used to determine the relationship between gut microbiota and serum metabolites.

## 3. Results

### 3.1. GABA Treatment Promotes Energy Consumption and Improves Glucose Metabolism

**Figure 1:**
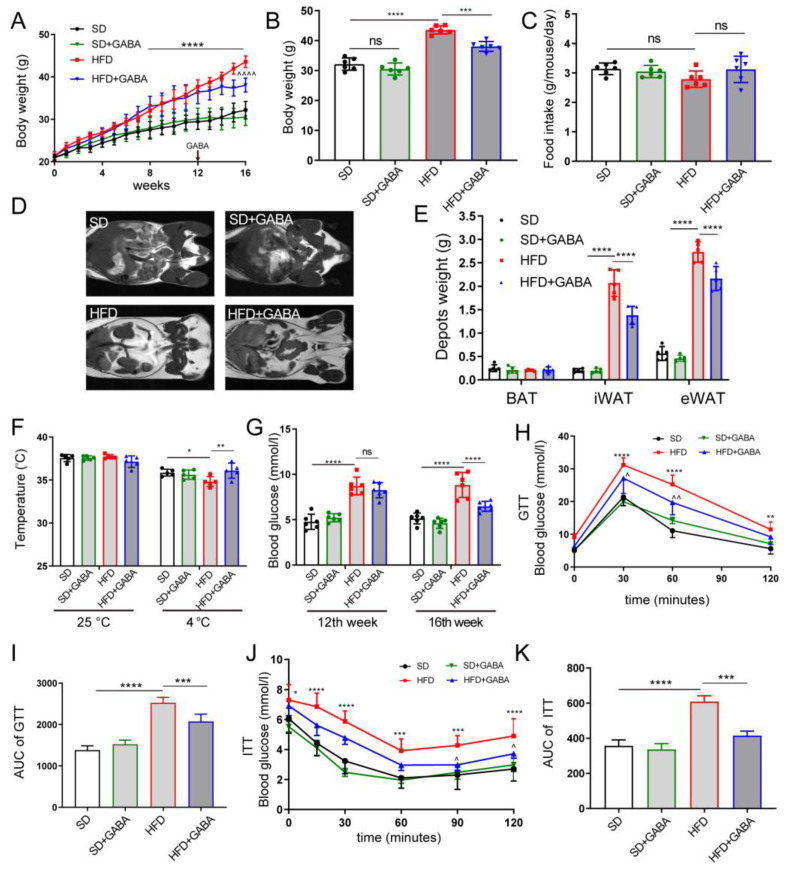
GABA treatment promotes energy consumption and improves glucose metabolism. Mice were administered SD (black), SD+GABA (green), HFD (red), or HFD+GABA (blue) treatment. (**A**) Changes in body weight curves of the mice over the 16 weeks of treatment. For SD+GABA and HFD+GABA group, 6 mg·mL^−1^ GABA was intervened at 12 weeks for a period of 4 weeks. (**B**) Body weight after the experiment. (**C**) Food intake of SD group, SD+GABA group, HFD group and HFD+GABA group. (**D**,**E**) MRI measurement of adipose tissue content (BAT, iWAT, and eWAT) and statistical analysis at the end of experiment. (**F**) Rectal temperature of mice in 25 °C (standard) or 4 °C (cold stimulation) for 180 min at the end of experiment. (**G**) Fast blood glucose concentrations at the 12th and the 16th week of the experiment. (**H**,**I**) Blood glucose concentrations during GTT (**H**), and the area under the GTT curve (**I**) at the end of experiment. (**J**,**K**) Blood glucose concentrations during ITT (**J**), and the area under the ITT curve (**K**) at the end of experiment. Data are presented mean  ±  standard deviation and were analyzed using a one-way or two-way ANOVA. * *p*  <  0.05, ** *p*  <  0.01, *** *p*  <  0.001, **** *p*  <  0.0001 compared to the SD group in (**A**,**B**,**F**–**K**). ^ *p*  <  0.05, ^^ *p*  <  0.01 and ^^^^ *p*  <  0.0001 compared to the HFD group in (**A**,**H**,**J**). *n*  =  5–6 for all groups. Ns, not significant. ANOVA, analysis of variance; BAT, brown adipose tissue; eWAT, epididymal white adipose tissue; GABA, Gamma-aminobutyric acid; GTT, glucose tolerance test; HFD, high-fat diet; ITT, insulin tolerance tests; iWAT inguinal white adipose tissue; SD, standard diet.

The HFD-induced obese mice were used to study the effect of GABA treatment on energy consumption. The body weights of mice in the HFD group significantly increased compared to those in the SD group during animal modeling (Figure 1A). HFD feeding for 16 weeks led to a significant increase in body weight (Figure 1B). Moreover, abdominal MRI revealed that the accumulation of epididymal and subcutaneous fat was significantly higher in HFD mice than in SD mice (Figure 1D,E). Meanwhile, four-week GABA intervention reduced weight gain and WAT accumulation in the HFD-fed mice for 12 weeks, while the weight and fat loss of the SD+GABA group mice were not apparent (Figure 1A–E). However, it is unclear how GABA reduced fat accumulation in obese mice, leading to weight loss.

Obesity develops when energy intake exceeds energy dissipation [25]. In this study, no significant difference was observed in the energy intake among HFD-fed mice (Figure 1C), which indicates that the effects of GABA treatment on body weight and obesity phenotype were not caused by reduced food consumption or energy intake. This, in turn, shows that GABA may have promoted energy expenditure in obese mice. After being kept in cold storage (at 4 °C) for 180 min, mice in the GABA group showed increased rectal temperatures compared to those in the HFD group (Figure 1F). This indicates that the GABA-treated mice dissipated more energy than those in the HFD group. After GABA treatment, the FBG in the HFD+GABA group mice significantly decreased compared to that in the HFD control mice (Figure 1G). In addition, the area under the curve (AUC) of the GTTs revealed that the HFD-fed mice had worse glucose tolerance than the SD mice and that GABA could restore glucose tolerance (Figure 1H,I). Similarly, regarding ITT analysis, the area under the curve values of GABA-treated mice were remarkably lower than those of the HFD control mice (Figure 1J,K). Overall, GABA treatment successfully promoted energy consumption and improved glucose tolerance and insulin resistance in obese mice.

### 3.2. GABA Promotes Energy Consumption through iWAT Beiging

**Figure 2:**
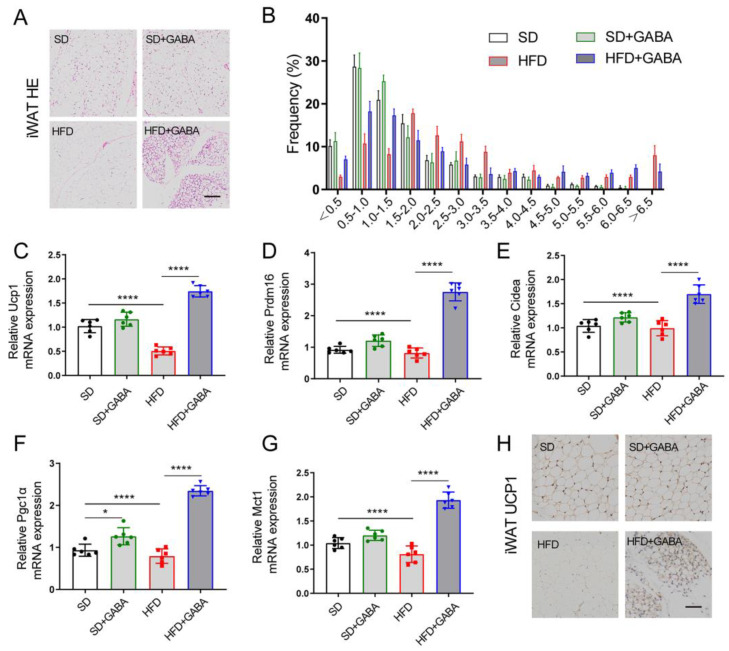
GABA treatment promotes energy consumption through iWAT beiging. (**A**) Representative H&E staining in the iWAT samples (Scale bars 100 μm). (**B**) Area of lipid droplets (*1000 μm^2^) was estimated using the Image J software. (**C**–**G**) Expression of thermogenic genes (including Ucp1, Prdm16, Cidea, Pgc1α, and Mct1) in the iWAT. (**H**) Representative UCP1 IHC staining of the iWAT (Scale bars, 50 μm). *n*  =  5–6 for all groups. β-actin as PCR reference gene in (**C**–**G**). Data are presented as mean ± standard deviation and were analyzed using a one-way or two-way ANOVA. * *p*  <  0.05 and **** *p*  <  0.0001. ANOVA, analysis of variance; iWAT inguinal white adipose tissue.

Non-trembling thermogenesis is mainly regulated by the activation of brown and/or beige adipocytes [26]. H&E staining revealed that the HFD increased fat deposition in iWAT tissue (Figure 2A). Meanwhile, GABA treatment reduced lipid accumulation in the HFD-induced obese mice (Figure 2A). Furthermore, the H&E staining of adipocyte area in GABA-treated mice revealed that adipocytes were smaller and fewer than those in the HFD control mice (Figure 2B). The expression of thermogenic genes in the BAT and iWAT was also evaluated. The results revealed that the mRNA expression of brown fat cell-specific genes (including Ucp1, Prdm16, Cidea, Pgc1a, and Mct1) in the iWAT was strongly activated in the GABA-treated mice (Figure 2C–G). Meanwhile, UCP1 antibody staining indicated an increased UCP1 protein expression in the iWAT in histological sections (Figure 2H). The H&E staining of BAT showed that the average volume of intracellular vacuoles in the HFD+GABA group was less than that in the HFD group, while similar between the SD and the SD+GABA group (Supplementary Figure S1A). Although the mRNA expression of Ucp1 and Mct1 in the BAT was increased in the SD+GABA group compared to the SD group, the thermogenic genes of BAT in the HFD+GABA group was not statistically higher than that in the HFD group (Supplementary Figure S1B–F). These results indicated that GABA played an important role in energy expenditure and iWAT beiging.

### 3.3. GABA Reduces Fat Inflammation and Restores Intestinal Structure in HFD Mice

**Figure 3:**
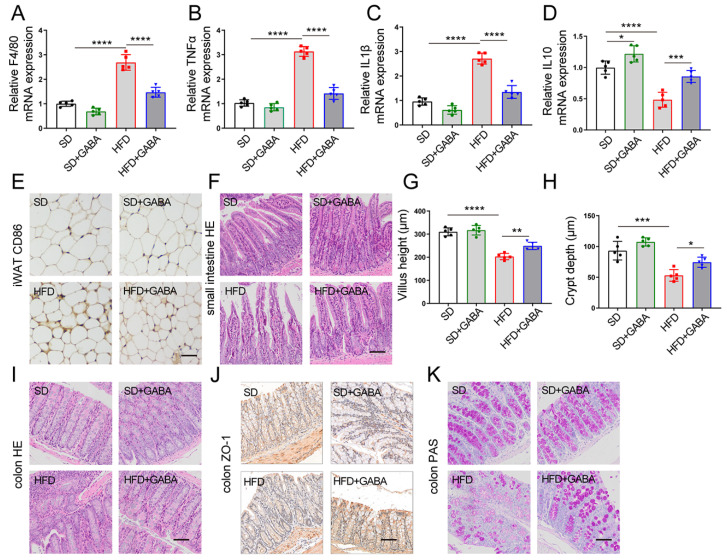
GABA treatment reduces fat inflammation and restored intestinal structure in HFD mice. (**A**–**D**) mRNA expression of inflammation-related factors (including F4/80, TNFα, IL 1β, and IL 10) in the iWAT. (**E**) Representative CD86 IHC staining of the iWAT (Scale bars, 50 μm). (**F**,**I**) Representative H&E staining in the small intestine and colon samples (Scale bars, 100 μm). (**G**,**H**) Villus height and crypt depth of the small intestine. (**J**) Representative ZO-1 IHC staining of the colon (Scale bars, 100 μm). (**K**) PAS staining of the colon (Scale bars, 50 μm). *n*  =  5 for all groups. Data are represented as means ± standard deviation and were analyzed by one-way ANOVA. * *p*  <  0.05, ** *p*  <  0.01, *** *p*  <  0.001 and **** *p*  <  0.0001. H&E, hematoxylin and eosin; IHC, immunohistochemistry; iWAT, inguinal white adipose tissue; PAS, periodic acid Schiff.

According to prior studies, HFD mice produce more macrophages as well as pro-inflammatory cytokines in the WAT than SD mice [27]. Furthermore, Nehemiah Cox et al. found that macrophages in the WAT respond to dietary fat intake and regulate fat storage in a paracrine manner [28]. Therefore, an analysis of the macrophage marker and cytokine expression after GABA treatment was conducted using quantitative PCR. Higher expression levels of macrophage marker F4/80 and pro-inflammatory cytokines (TNFα and IL1β) and lower expression of the anti-inflammatory cytokine IL10 were observed in the iWAT of HFD mice than in that of the SD group (Figure 3A–D). Notably, obesity significantly increased the number of CD86-positive M1 macrophages in adipose tissues (Figure 3E), whereas GABA treatment reduced inflammation and proinflammatory M1 macrophages in the iWAT of the HFD group (Figure 3A–E).

Owing to the general association of obesity with damaged intestinal integrity [29], the morphology of small intestine and colon was observed in this study. H&E staining showed that villus height of the small intestine was less and crypt depth was shallower in the HFD group than those in the SD group, which was partially improved by GABA treatment (Figure 3F–H). The major tight junction protein ZO-1 plays an important role in maintaining intestinal integrity in the colon. According to IHC analysis, HFD administration reduced ZO-1 expression, which was significantly increased by GABA treatment (Figure 3J). Furthermore, PAS staining demonstrated that HFD administration reduced the number of goblet cells and glycogen accumulation in the colon, which was increased by GABA treatment (Figure 3K). Overall, a major result of GABA treatment in HFD mice was the reduction in fat inflammation and the enhancement of intestinal barrier integrity.

### 3.4. GABA Modulates the Composition of Gut Microbiota

**Figure 4:**
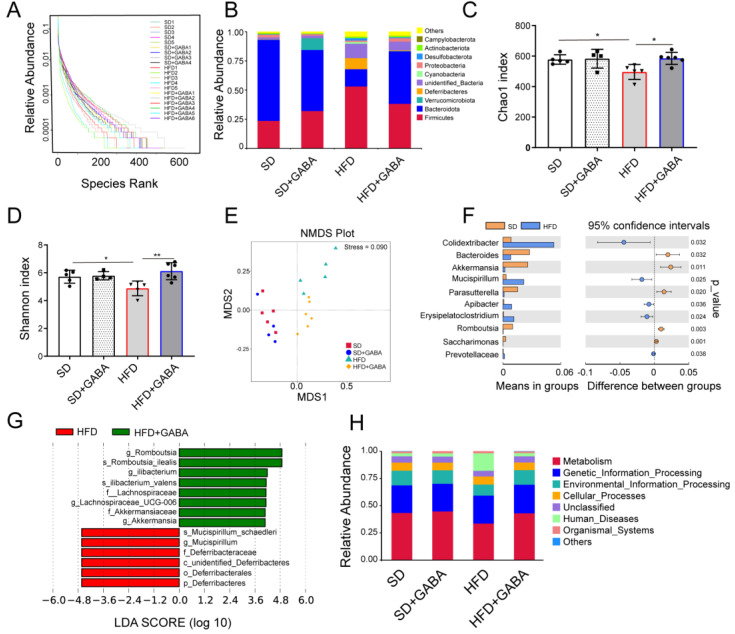
GABA modulates the composition of gut microbiota. (**A**) Relative abundance of intestinal flora in the four experimental groups. (**B**) Phylum level analyses were based on OTU classification; “others” indicates OTUs that were not classified at the phylum level. (**C**,**D**) Gut microbiota richness, as indicated by the Chao1 and Shannon indices. (**E**) NMDS plots for metabolomics analysis (Stress = 0.09). (**F**) The *t*-test of differential species abundance under each level between the SD group and HFD group. (**G**) Comparison of microbial variations, using LEfSe (LDA Effect Size) analysis, between the HFD and HFD+GABA groups. (**H**) Relative abundance of Tax4Fun function annotations. LEfSe, linear discriminant analysis effect size. *n*  =  4–6 for all groups. OTU, operational taxonomic unit. * *p*  <  0.05 and ** *p*  <  0.01.

To investigate whether the anti-obesity and beiging of iWAT effects of GABA are related to the gut microbiota, 16S rRNA gene sequencing of the gut microbiota was conducted. On the rank charts, the HFD group had a smaller lateral range than the SD group, indicating less species richness (Figure 4A). Compared to that in the HFD group, the curve in the GABA-treated HFD group was flatter, indicating that the species were more equally distributed (Figure 4A). More OTUs were identified in the GABA-treated group than in the HFD control group, which indicated that the former had a higher microbiota abundance (Figure 4B). Phylum level analysis of the gut microbiota indicated that HFD administration significantly reduced Bacteroidetes and increased Firmicutes abundance relative to that in the SD group, whereas GABA treatment reversed these changes (Figure 4B). Both the Chao 1 and Shannon indices revealed significant differences in the alpha diversity among the SD, HFD and GABA groups (Figure 4C,D). Compared to that in the SD group, the microbial diversity was low in the HFD mice, which has previously been shown to be strongly associated with adiposity [14]. These results indicated that the gut microbial community of HFD mice was significantly affected by GABA treatment.

Beta diversity analysis, using NMDS, revealed that GABA influenced the gut microbiota composition in HFD mice (Figure 4E). A *t*-test of species differences at the genus level between the SD and HFD groups showed that HFD administration increased the abundance of harmful bacteria (including Colidextribacter, Mucispirillum, and Erysipelatoclostridium) while reducing that of anti-inflammatory bacteria (Bacteroides and Akkermansia) compared to that in the SD group (Figure 4F). Additionally, the LEfSe analysis demonstrated significant species differences between the HFD and HFD+GABA groups. Based on statistical analysis, GABA treatment increased the relative abundance of Ileibacterium, Akkermansia, and Romboutsia while reducing that of Deferribacteres and Mucispirillum (Figure 4G). In addition, Tax4Fun [30] was used to predict the relative abundance of functional categories from databases such as the Kyoto Encyclopedia of Genes and Genomes (KEGG). Our results showed that the pathways involved in metabolism and genetic information processing were upregulated in the fecal microbiome of the GABA group, whereas those involved in human diseases were upregulated in the fecal microbiome of the HFD control group (Figure 4H), indicating the different functions of the fecal microbiota between the SD and HFD groups with or without GABA treatment. Overall, these results suggest that the composition and function of gut microbiota are influenced by both an HFD and GABA.

### 3.5. Gut Microbiota Mediates the Effect of GABA on iWAT Beiging

**Figure 5:**
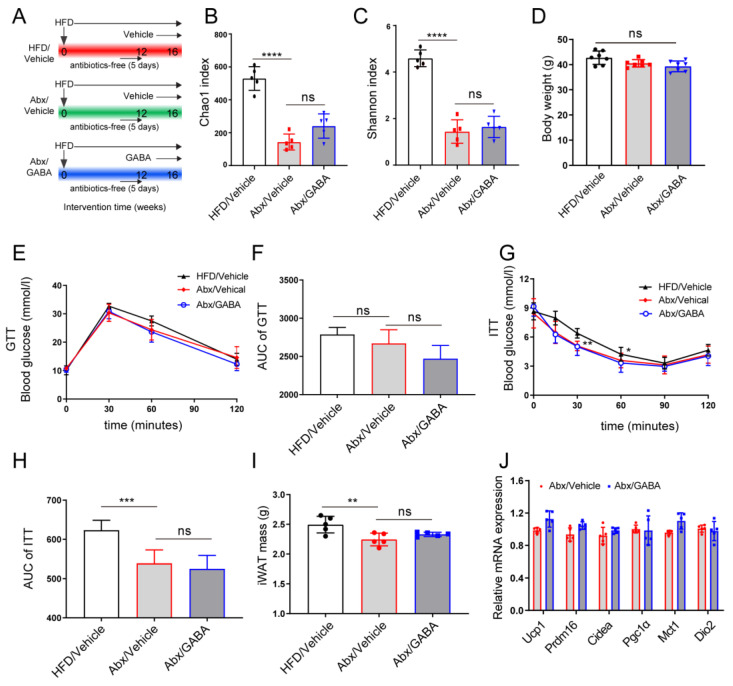
Gut microbiota mediates the effect of GABA on the beiging of iWAT. Intestinal microbiota-free HFD-fed mice were treated daily with the vehicle or GABA for 4 weeks. (**A**) Flow chart of animal experiments. (**B**,**C**) Gut microbiota richness, as indicated by the Chao1 and Shannon indices. (**D**) Body weight after the experiment. (**E**,**F**) GTT curve and the AUC analysis. (**G**,**H**) ITT curve and the AUC analysis. (**I**) Tissue weights of iWAT. (**J**) Expression of thermogenic genes (including Ucp1, Prdm16, Cidea, Pgc1α, Mct1, and Dio2) in the iWAT. Abx, antibiotics. *n*  =  5–7 for all groups. Data are represented as means ± standard deviation and were analyzed by one-way or two-way ANOVA. Ns, not significant. ** *p*  <  0.01, *** *p*  <  0.001 and **** *p*  <  0.0001. GABA, Gamma-aminobutyric acid; HFD, high-fat diet; NMDS, non-metric multidimensional scaling; OTU, operational taxonomic unit.

To further validate the role of gut microbiota in the therapeutic effect of GABA on WAT beiging in HFD mice, antibiotics were used to eliminate the gut microbiota of HFD model mice, following which GABA was administered for four weeks (Figure 5A). Gut microbiota analysis showed that the species richness (measured using Chao1 and Shannon Indexes) statistically decreased after antibiotic intervention and that it was not significantly modified between the Abx/HFD group and Abx/GABA group (Figure 5B,C). Without microbiota, the GABA treatment only slightly reduced the weights of obese mice (Figure 5D) and could not ameliorate glucose metabolism or insulin sensitivity (Figure 5E–H). Compared to that in the Abx/vehicle group, the iWAT did not decrease in the Abx/GABA group (Figure 5I). Importantly, the mRNA expression of brown fat cell-specific genes (including Ucp1, Prdm16, Cidea, Pgc1a, Mct1, and Dio2) in the Abx/GABA group showed no statistically significant increase (Figure 5J). These data indicate that the effects of GABA on glucose metabolism and fat beiging were dependent on the gut microbiota.

**Figure 6:**
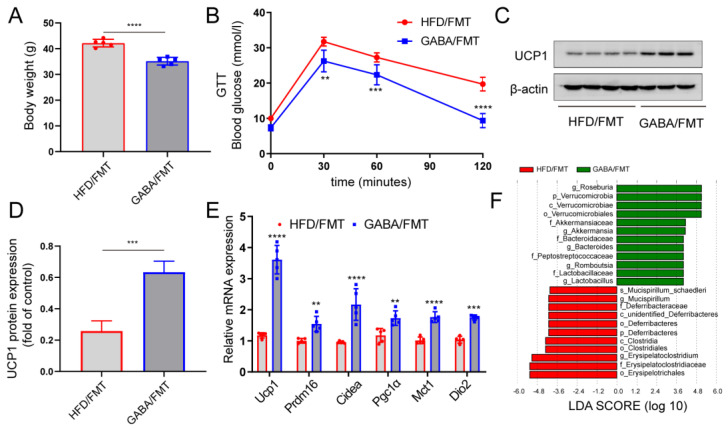
FMT is sufficient to induce the beiging of iWAT caused by GABA. Intestinal microbiota-free HFD-fed mice were colonized by fecal matter from either HFD-fed mice or GABA-treated mice for one week. (**A**) Body weight after the FMT experiment. (**B**) GTT curve of the HFD/FMT and GABA/FMT groups. (**C**,**D**) UCP1 protein expression in the iWAT was detected via immunoblot analysis. β-actin was used as the internal reference. (**E**) Relative mRNA expression of thermogenic genes in the HFD/FMT and GABA/FMT groups. (**F**) Histograms of LDA scores identifying taxa differentially represented between the HFD/FMT and GABA/FMT groups (LDA score > 2). Data are presented as mean ± standard deviation and were analyzed using a two-tailed Student’s *t*-test or two-way ANOVA. ** *p*  <  0.01, *** *p*  <  0.001 and **** *p*  <  0.0001 compared to HFD/FMT group. ANOVA, analysis of variance; FMT, fecal microbiota transplantation; GABA, Gamma-aminobutyric acid; GTT, glucose tolerance test; HFD, high fat diet; iWAT inguinal white adipose tissue; LDA, linear discriminant analysis.

To further test this hypothesis, the FMT experiment was conducted, fecal matter from HFD mice treated with or without GABA was transplanted into the HFD mice treated with antibiotics. Compared with the HFD/FMT group, GABA treatment restored metabolic dysfunction in the GABA/FMT group (Figure 6A,B). That is, the obese mice of the GABA/FMT group lost weight and displayed improved glucose tolerance. Transplantation of the fecal matter from GABA-treated mice into sterile HFD mice promoted the expression of UCP1 protein and thermogenic genes in iWAT (Figure 6C–E). LEfSe analysis showed that GABA/FMT increased the relative abundance of Akkermansia, Romboutsia, and Lactobacillus, while it reduced the abundance of Erysipelatoclostridium and Deferribacteres (Figure 6F). Overall, these findings further demonstrate the role of gut microbiota in the effects of GABA on WAT beiging and the improvement of metabolic diseases.

### 3.6. Effect of GABA on Serum Metabolites

**Figure 7:**
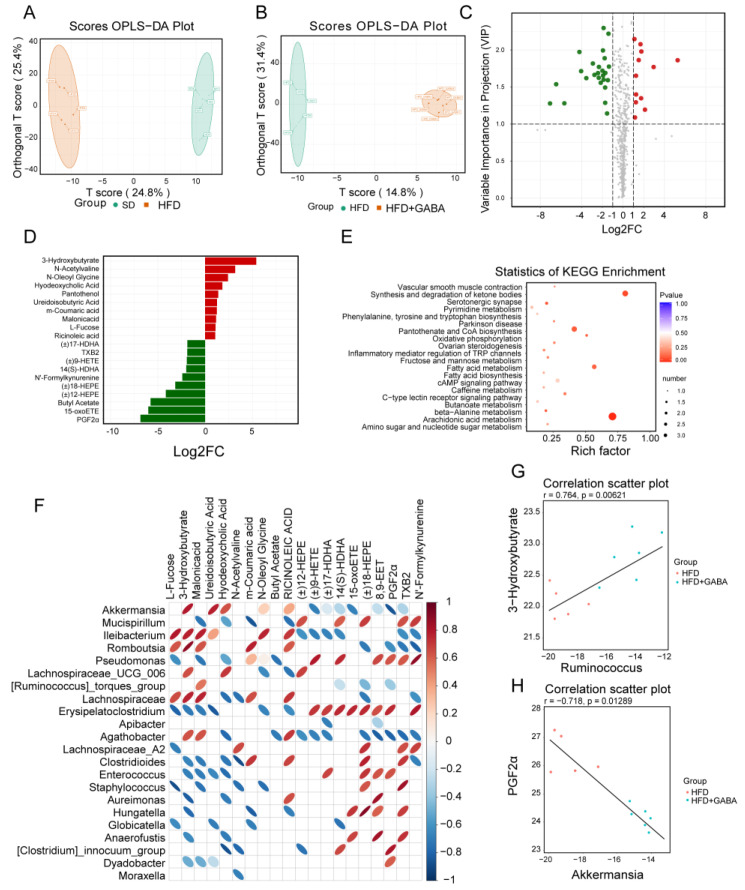
Bacterial metabolites affect the regulation of GABA on fat beige. (**A**,**B**) OPLS-DA score plot of metabolite profiles. (**C**) Volcano plot of metabolites between the HFD and HFD+GABA groups. The green dots represent downregulated differentially expressed metabolites, the red dots represent upregulated differentially expressed metabolites, and the gray ones represent detected but not significant metabolites. (**D**) Histograms of LDA scores identifying the taxa differentially represented between HFD and HFD+GABA groups (LDA score > 2). (**E**) Statistics of KEGG enrichment and classification. (**F**) Correlation diagram between the top 20 differential metabolites and intestinal microbes in the HFD and HFD+GABA groups. Each row represents a type of microorganism, and each column represents a metabolite. The red ellipse indicates a positive correlation, and the blue ellipse indicates a negative correlation. The greater the absolute value of the correlation, the thinner the ellipse. A blank grid indicates a significant *p*-value > 0.05. (**G**,**H**) Spearman correlation analysis between 3-hydroxybutyrate and Ruminococcus (**G**) and PGF2α and Akkermansia (**H**). OPLS-DA, orthogonal partial least squares discriminant analysis.

Intestinal microbiota is known to shape the metabolic pathways and obesity in the host; metabolomic studies are continuously expanding our knowledge regarding the impact of microbiota on metabolic diseases [31]. In this study, the non-targeted metabolomic profiling of more than 500 metabolites was conducted on the plasma from SD and HFD mice treated with or without GABA. First, an orthogonal partial least squares discriminant analysis (OPLS-DA) was performed to analyze the metabolomics data. The OPLS-DA model indicated significant metabolic variations between the SD and HFD groups as well as the HFD and HFD+GABA groups (Figure 7A,B). Next, heatmap analyses of metabolites were performed. Metabolites with fold changes ≥ 2 and a variable important in projection values of ≥1 were selected (Figure 7C). Heatmap analyses revealed there was no difference in the expression of most metabolites between the HFD and HFD+GABA groups and that the reduction in the levels of differentially expressed metabolites was higher than the increase in their levels (Figure 7C).

The LEfSe analysis showed that GABA treatment resulted in significant metabolic variations compared to those in the HFD control group (Figure 7D). As previously mentioned, obesity promotes an increase in the levels of oxidized lipids [32]. Among the 37 metabolites with statistically significant differences in serum levels, several oxidized lipids, including (±)17-HDHA, (±)18-HEPE, (±)9-HETE, 12-EET, 14(S)-HDHA, and 15-oxoETE, were enriched in the HFD control group, whereas GABA treatment significantly reduced their concentrations but increased the levels of 3-hydroxybutyric acid and hyodeoxycholic acid (Figure 7D). Next, metabolomics pathway analysis was used to explore the metabolic pathways that may be affected by GABA treatment. As shown in Figure 7E, KEGG enrichment analysis results showed that the metabolic pathways largely involving arachidonic acid and fatty acid metabolism and pantothenate and CoA biosynthesis, which are important for understanding the effect of GABA on obesity. Among these, arachidonic acid metabolism was identified as the most important pathway. According to these results, the HFD-induced serum metabolism abnormalities were effectively reversed by GABA treatment.

### 3.7. Potential Relationships between Serum Metabolites and the Gut Microbiota

To intuitively reflect the correlation between the expression of differential microbiota and metabolites, Spearman correlation analysis was conducted. Correlation data among the top 20 differential metabolites and microbiota were extracted to draw a heat map. As shown in Figure 7F, some bacteria (including Akkermansia, Romboutsia, Ileibacterium, and Lachnospiraceae_UCG_006) were negatively correlated, while other bacteria (including Mucispirillum, Pseudomonas, and Erysipelatoclostridium) were positively correlated with the levels of oxidized lipids, such as PGF2α. Additionally, 3-hydroxybutyrate was positively correlated with Romboutsia (Figure 7G) and PGF2α was negatively correlated with Akkermansia (Figure 7H). These relationships suggest that the gut microbiota could affect serum metabolite levels. Metabolomics analysis of the gut microbiota and metabolites may provide direction for further research into the pathogenesis of obesity and the mechanism of GABA treatment.

## 4. Discussion

The incidence of obesity and related metabolic abnormalities has been increasing rapidly in recent years; therefore, there is an urgent need to find new treatment strategies to prevent obesity. Several studies have shown that increasing the heat production in WAT and BAT may be an effective strategy for exploring obesity treatments. In mainstream metabolic disease research, several genetic mouse models (such as ob/ob and db/db mice) and HFD-induced obesity mouse models have been used [33]. Here, a diet-induced obese mouse model was used to mimic the development of human obesity. Studies have reported that a reduced thermogenic activity of BAT or the lack of a WAT beige effect can lead to diet-induced obesity [5,26]. Here, HFD mice showed significantly reduced levels of beige cells in the iWAT, which indicated that obesity led to fat metabolism and differentiation barriers. This study confirmed that GABA treatment could improve the metabolic syndrome by regulating the beiging of iWAT.

GABA is widely concerned because of its good physiological function and application prospect and has been used in food and medicine. In recent metabolism-related studies, GABA has mainly been assessed regarding the pathogenesis of type 1 diabetes [18,34]. In fact, GABA receptors are expressed in many tissues, including intestinal, hepatic, and adipose [35,36]. Our previous study revealed that GABA can improve glucose metabolism by reducing β-cell dedifferentiation [17], although the mechanisms of weight loss and increased insulin sensitivity remain unclear. Here, the effect of GABA on weight loss in HFD mice was investigated, demonstrating that GABA plays an important role in the beiging of iWAT. One surprising discovery of this study was that GABA promoted the beiging of iWAT rather than BAT activation in HFD mice, while the effect was not evident in the SD+GABA mice (Figure 2C–H and Supplementary Figure S1B–F). Therefore, understanding the potential mechanism by which GABA promotes the beiging of iWAT may provide new targets for the prevention and treatment of metabolic diseases and could provide an alternative drug choice to help obese patients to lose weight.

Fecal microbiota analysis showed that GABA treatment improved the gut microbiota dysbiosis induced by an HFD. Consistent with previous reporting, obesity was associated with a relative increase in Firmicutes abundance at the expense of Bacteroidetes [37], while GABA treatment significantly reduced Firmicutes and increased abundance in HFD mice (Figure 4B). GABA treatment increased the relative abundance of Akkermansia and Romboutsia, which were negatively associated with the body weight and beiging of iWAT. As is well known, Akkermansia reduces adiposity and improves glucose homeostasis in HFD mice [38]. In the small intestine, Romboutsia is a natural organism that can utilize carbohydrates [39]. Therefore, Romboutsia may also be considered a candidate genus to predict and treat obesity and related metabolic disorders.

As the metabolome comprises both human and microbial metabolic activities, we further examined the serum metabolites. The analysis of non-targeted serum metabolomics showed that GABA treatment influenced the metabolism of lipids and ketone bodies, particularly by altering the concentrations of oxidized lipids, which were found to be the hub of gut microbiota regulating fat inflammation and metabolism. In the present study, GABA treatment was found to improve the gut microbiota composition of HFD mice. The correlation analysis of serum metabolites and gut microbiota revealed that the abundance of bacteria (phylum Verrucomicrobia; genera Akkermansia, Roseburia, and Lactobacillus) increased by GABA treatment correlated negatively with the levels of oxidized lipids. Moreover, 3-hydroxybutyrate showed a positive correlation with the genus Akkermansia (Figure 7F). These findings indicate that gut microbes have an impact on serum metabolism. Moreover, clearing gut microbiota with antibiotics was found to significantly inhibit the effects of GABA on the beiging of iWAT in obese mice. After gavaging GABA-treated mouse feces into sterile HFD mice, the thermogenic gene levels in iWAT were found to significantly increase. These results showed that GABA treatment improved the gut microbiota composition of obese mice, thereby increasing their beige fat content and ultimately improving their metabolic levels. If these results could be further confirmed in clinical trials, GABA could be used to treat obesity-related metabolic disorders. Overall, the potential biomarkers related to gut microbiota provided useful information for understanding the effects of GABA on obesity and strengthened its therapeutic value for treating obesity.

The results of this study revealed that gut microbiota mediated the mechanism of GABA-induced WAT beiging. Previous studies have proposed several mechanisms of fat beiging, such as the β-AR signaling pathway [40]. Although the activation of BAT mostly depends on β-AR signaling, it has been reported that the beiging of iWAT may not be related to β-AR signaling [8,14]. Recent studies have demonstrated that interactions between the host and gut microbiota can affect many aspects of energy metabolism [41]. The microbiome has also been shown to influence cold-induced fat formation and regulate metabolic diseases [42]. Interestingly, the mechanism by which GABA altered the composition of gut microbes in this study was similar to the mechanisms identified in previous studies. This view supports the causal role of microbes in WAT inducement [10,14]. The transplantation of fecal matter from GABA-treated HFD mice into sterile HFD mice promoted the beiging of iWAT, indicating that GABA-induced WAT beiging requires the participation of gut microbiota. However, we cannot rule out the possibility that certain metabolic changes in the sterile mice may have been caused by antibiotics.

This study still has some limitations. First, this study cannot demonstrate that GABA exerts weight loss effects through a single bacterial change. Second, the study has not identified which metabolite changes directly mediate beige adipocyte reconstruction of GABA. More studies are needed to further explore the regulatory relationship between metabolites and white adipocyte browning. In addition, these findings were obtained in animals and require further confirmation by clinical studies.

In summary, this study showed that GABA promoted iWAT beiging via the gut microbiota; however, the molecular mechanism of the gut-fat axis remains unclear. Further research on the transplantation of specific gut microbiota or metabolites in sterile mouse models may provide an insight into this aspect.

## 5. Conclusions

In summary, this study demonstrated that GABA reduced obesity by reducing WAT deposition and increasing iWAT beiging. Additionally, variations in serum metabolism caused by the gut microbiota contributed to a better understanding of the potential mechanisms underlying the anti-obesity effects of GABA. Furthermore, a key finding of our study suggests that GABA may be a potential anti-obesity drug and that the gut microbiota may be a potential target for GABA therapy.

## Figures and Tables

**Table 1:** Sequences of the gene-specific primers.

Gene	Forward Primer (5′-3′)	Reverse Primer (5′-3′)
Ucp1	GATGGTGAACCCGACAACTTCCGAAGTG	TTCACCTTGGATCTGAAGGCGGACTTTGG
Prdm16	TCTACATTCCTGAAGACATTCCAATCCCACCA	TGTATCCGTCAGCATCTCCCATCCAAAGTC
Cidea	CGAGTTTCAAACCATGACCGAAGTAGCC	CTTACTACCCGGTGTCCATTTCTGTCCC
Pgc1α	GACAGGTGCCTTCAGTTCACTCTCAG	AGCAGCACACTCTATGT-CACTCCATACAG
Mct1	AGGTCCTATCAGCAGTATCT	AGTTCCTGCACCGTGTTACA
Dio2	TACAAACAGGTTAAACTGGGTGAAGATGCTC	GAGCCTCATCAATGTATACCAACAGGAAGTC
F4/80	GGATGTACAGATGGGGGATG	CATAAGCTGGGCAAGTGGTA
TNFα	TCCCTCTCATCAGTTCTATGGCCCA	CAGCAAGCATCTATGCACTTAGACCCC
IL1β	CGGCACACCCACCCTG	AAACCGTTTTTCCATCTTCTTCT
IL10	GCTCTTACTGACTGGCATGAG	CGCAGCTCTAGGAGCATGTG
β-actin	GGCACCACACCTTCTACAATG	GTGGTGGTGAAGCTGTAGCC

Abbreviations: Ucp1, uncoupling protein 1; Prdm16, PR-domain containing 16; Cidea, cell death-inducing DNA fragmentation factor α-subunit effector 1; Pgc1α, proliferator-activated receptor-coactivator-1α; Mct1, monocarboxylate transporter isoform 1; Dio2, deiodinase 2; TNFα, tumor necrosis factor α; IL, interleukin.

## Data Availability

Not applicable.

## Supplementary Materials

The following supporting information can be downloaded at: https://www.mdpi.com/article/10.3390/nu15020456/s1, Figure S1: Histomorphological and thermogenic genes change in BAT. (A) Representative H&E staining in the BAT samples (Scale bars, 100 μm). (B–F) Thermogenic genes (including Ucp1, Prdm16, Cidea, Pgc1α and Mct1) expression in the BAT.
