# Supplementary figures and images for: Gamma-Aminobutyric Acid Promotes Beige Adipocyte Reconstruction by Modulating the Gut Microbiota in Obese Mice
Source: Nutrients. 2023 Jan 15;15(2):456. doi: 10.3390/nu15020456 (PMC9864545; doi:10.3390/nu15020456)

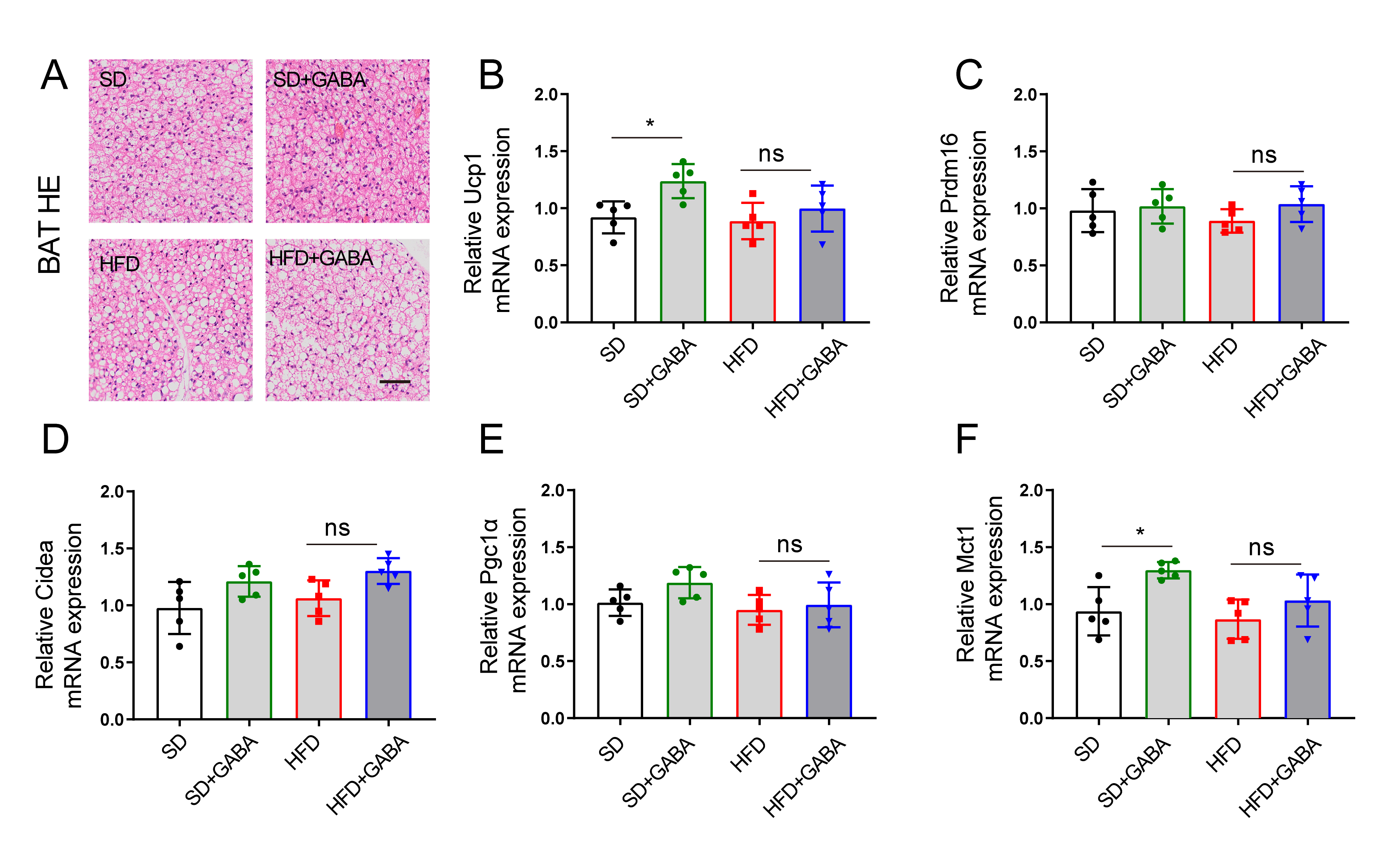

Supplement: Supplementary file 1 [file nutrients-15-00456-s001.zip › nutrients-2076062-supplementary.tif]
